# The association of pretreatment low skeletal muscle mass with chemotherapy dose‐limiting toxicity in patients with head and neck cancer undergoing primary chemoradiotherapy with high‐dose cisplatin

**DOI:** 10.1002/hed.26919

**Published:** 2021-10-29

**Authors:** Sandra I. Bril, Abrahim Al‐Mamgani, Najiba Chargi, Peter Remeijer, Lot A. Devriese, Jan Paul de Boer, Remco de Bree

**Affiliations:** ^1^ Department of Head and Neck Surgical Oncology, UMC Utrecht Cancer Center University Medical Center Utrecht Utrecht The Netherlands; ^2^ Department of Radiation Oncology Netherlands Cancer Institute/Antoni van Leeuwenhoek Amsterdam The Netherlands; ^3^ Department of Medical Oncology, UMC Utrecht Cancer Center University Medical Center Utrecht Utrecht The Netherlands; ^4^ Department of Medical Oncology Netherlands Cancer Institute/Antoni van Leeuwenhoek Amsterdam The Netherlands

**Keywords:** antineoplastic agents, body composition, head and neck neoplasms, radiotherapy, treatment outcome

## Abstract

**Background:**

Low skeletal muscle mass (SMM) is an adverse prognostic factor for chemotherapy dose‐limiting toxicity (CDLT). In patients with locally advanced head and neck squamous cell carcinoma (HNSCC) undergoing chemoradiotherapy (CRT), low SMM is a predictor for CDLT. We aimed to validate these findings.

**Methods:**

Consecutive LA‐HNSCC patients treated with primary CRT with high‐dose cisplatin were retrospectively included. SMM was measured on pre‐treatment CT‐imaging. A cumulative cisplatin dose below 200 mg/m^2^ was defined as CDLT.

**Results:**

One hundred and fifty three patients were included; 37 (24.2%) experienced CDLT, and 84 had low SMM (54.9%). Patients with low SMM experienced more CDLT than patients with normal SMM (35.7% vs. 10.1%, *p* < 0.01). Low SMM (OR 3.99 [95% CI 1.56–10.23], *p* = 0.01) and an eGFR of 60–70 ml/min (OR 5.40 [95% CI 1.57–18.65], *p* < 0.01) were predictors for CDLT.

**Conclusion:**

Pre‐treatment low SMM is associated with CDLT in LA‐HNSCC patients treated with primary CRT. Routine SMM assessment may allow for CDLT risk assessment and treatment optimization.

## 1 INTRODUCTION

Locally advanced head and neck squamous cell carcinoma (LA‐HNSCC) is preferably treated with concomitant chemoradiotherapy (CRT) with cisplatin, with or without prior surgery.^1^ The standard‐of‐care cisplatin regimen consists of 3 three‐weekly courses of high‐dose cisplatin at a dose of 100 mg/m^2^ body surface area (BSA), with a cumulative dose of 300 mg/m^2^ BSA cisplatin.^2^ The addition of high‐dose cisplatin chemotherapy to radiotherapy treatment improves locoregional disease control and results in a 6.5% increase in five‐year overall survival.^3^ Large prospective trials and retrospective studies show that a higher cumulative dose is associated with better survival rates.^4^, ^5^, ^6^, ^7^

The addition of cisplatin also results in a significant increase in the toxicity of treatment, such as acute nephrotoxicity, bone marrow depression, or severe nausea and vomiting, which cause treatment delay, dose reduction, and treatment cessation as well as decreased quality of life.^2^, ^8^ Approximately 30% of patients experience chemotherapy dose‐limiting toxicity (CDLT) and are unable to complete full treatment.^9^ There are several contraindications for the use of high‐dose cisplatin, such as a decreased renal function, severe hearing loss, and poor WHO functional status. Nevertheless, even in the absence of these contraindications, still 30% of patients experience CDLT in daily clinically practice, which currently cannot be identified in advance. Therefore, there is a clinical need for additional predictive characteristics or biomarkers to accurately identify LA‐HNSCC patients at high risk of CDLT from cisplatin.

In recent years, radiologically identified sarcopenia or low skeletal muscle mass (SMM) has been identified as a novel predictive and prognostic factor in patients with cancer. Pre‐treatment low SMM is associated with chemotherapy‐induced toxicity and CDLT in patients with a variety of cancer types, including lung, renal cell, colorectal, and breast cancer.^10^, ^11^ Several risk factors for low SMM are known, including malnutrition, immobilization, and chronic illnesses including cancer.^12^ In HNSCC, malnutrition at diagnosis is very common, and several retrospective studies report an incidence of approximately 50% of low SMM in HNSCC patients.^9^, ^13^, ^14^, ^15^

Recent retrospective studies in LA‐HNSCC patients also concluded that pre‐treatment low SMM was a significant predictor of CDLT in patients treated with CRT with platinum‐based chemotherapy.^9^, ^16^ The purpose of this study was to investigate and validate the predictive value of low SMM on CDLT in a larger cohort of LA‐HNSCC patients, treated with standard‐of‐care treatment with primary CRT with high‐dose cisplatin.

## 2 METHODS

This study was performed as a secondary analysis of a prior retrospective study; all body composition data and SMM measurements were newly acquired.^6^ All data were used in a coded fashion. Because of the retrospective nature of this study, formal informed consent or medical ethical board approval was waived at the time of the inception of this study. This research was conducted in accordance with the Declaration of Helsinki and all subsequent legislation.

### 2.1 Patient and study design

All patients were treated at the Netherlands Cancer Institute in Amsterdam, The Netherlands, with curative intent. Between January 2008 and December 2015, all 279 consecutive patients with histologically proven squamous cell carcinoma of the oropharynx, hypopharynx, or larynx who were eligible for concomitant primary CRT with 3 three‐weekly courses of high‐dose cisplatin courses at 100 mg/m^2^ BSA were identified. Patients who were not treated with cisplatin for any reason, and patients who received cisplatin in another regimen such as weekly cisplatin or carboplatin were excluded. Patients with a significantly decreased renal function, defined as an eGFR <60 (chronic kidney disease stage 3A or higher) were deemed high‐dose cisplatin unfit per national guidelines, and were offered different treatment by their consultant head and neck surgeon or oncologist. Patients without recent CT or MRI scans (less than 3 months) of the head and neck area prior to TL were excluded. Patients who had severe dental artifacts at the level of C3 that impeded accurate assessment of SMM were also excluded. Relevant clinical information such as weight, stature, body mass index (BMI), smoking, AJCC stage according to the 7th AJCC staging manual, and outcome data were retrieved from medical records. The Adult Comorbidity Evaluation index (ACE‐27) was used to measure comorbidities.^17^ In oropharyngeal cancer, HPV status was assessed by p16 staining, followed by high‐risk HPV PCR for confirmation. Survival data were collected until February 2017. Because of a known better prognosis of HPV‐related oropharyngeal cancer, those patients were excluded from survival analysis.

### 2.2 Chemotherapy dose‐limiting toxicity

CDLT was defined as any toxicity resulting in a cumulative cisplatin dose of less than 200 mg/m^2^. This could be because of a chemotherapy dose‐reduction of ≥50% (e.g., due to neutropenia or nephrotoxicity) after the first cycle of treatment, a postponement of treatment of ≥4 days (e.g., in the case of bone marrow suppression) resulting in termination of a cycle combined with a dose‐reduction, or a definite termination of chemotherapy after the first cycle of therapy. The aim was to complete all three cycles, but if treatment tolerance was perceived to be low, two full cycles of high‐dose cisplatin were accepted as adequate treatment. The decision for a chemotherapy dose reduction and/or chemotherapy termination was made at the treating oncologist's discretion.

### 2.3 CT image acquisition

As part of radiotherapy planning, pre‐treatment head and neck CT imaging in radiation mold was performed in all patients. Patients were immobilized in supine treatment position in a custom‐made head‐and‐neck mask. For planning, contrast‐enhanced 3‐mm slides, CT‐scan simulation was performed in all patients. All patients were treated with intensity‐modulated radiotherapy (IMRT) or volumetric modulate arc therapy (VMAT). The radiation treatment consisted of 46 Gy of elective irradiation to both sides of the neck (levels II–IV in case of node‐negative neck and levels II–V in case of cervical lymph node metastases), followed by a boost of 24 Gy in 12 fractions to the primary tumor and the involved nodes in case of node‐positive disease, to a total dose of 70 Gy.

### 2.4 Image evaluation

**FIGURE 1:**
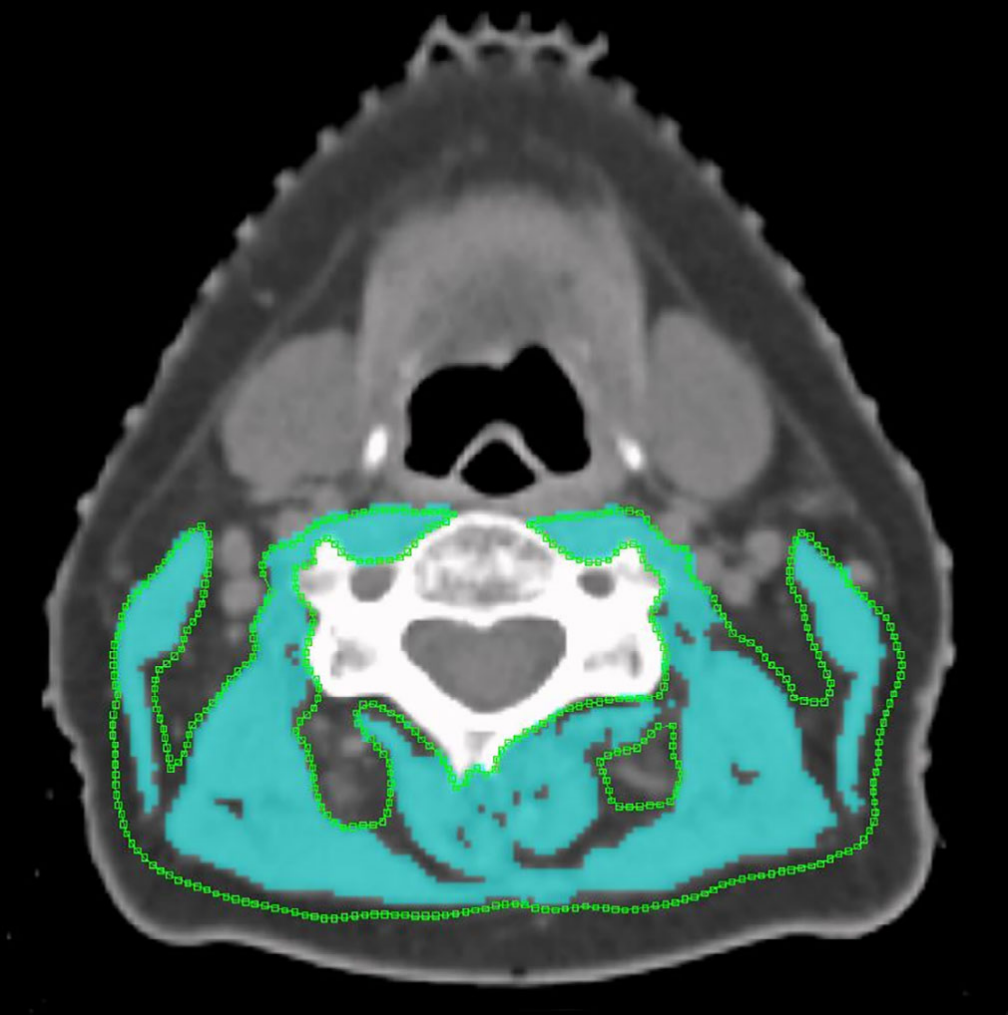
Skeletal muscle area segmentation at the level of C3 using the WorldMatch software program

Measurement of SMM was performed at the level of C3 according to a method previously described by Swartz et al.^18^ In brief, a single axial CT‐slide at level C3 was selected using a standard procedure: the first slide to completely show the entire vertebral arc when scrolling through the C3 vertebra from caudal to cephalic direction was selected. Skeletal muscle tissue was identified using Hounsfield unit (HU) ranges settings from −29 to +150 HU, to avoid overestimation of skeletal muscle area and to exclude fatty tissue (which has an HU value below −30).^19^ The outer contours of the sternocleidomastoid and paravertebral muscles were traced manually (Figure 1) using the Worldmatch Research Software Package, an in‐house software package designed for image evaluation, registration, and delineation for radiotherapy. The cross‐sectional muscle area (CSMA) at the level of C3 was calculated as the sum of the delineated areas of the paravertebral muscles and both sternocleidomastoid muscles within HU ranges of −29 to +150 in cm^2^. All CT slides were analyzed by a single researcher (S.B.). The CSMA at the level of C3 was then normalized for stature to calculate a cervical skeletal muscle index (CSMI).^20^

### 2.5 Statistical analysis

All analyses were performed using SPSS version 25.0 (SPSS Inc., Chicago, IL, USA). Continuous data are represented as mean ± SD. Categorical data are represented as the number and percentage of total. The optimum SMM cut‐off value based on CDLT was obtained using the optimal point in a receiver optimum stratification for binary outcomes (in this study, the occurrence of CDLT). The Fisher's exact test, Pearson Chi square test, independent sample *t*‐test, and Mann–Whitney U test were used for comparisons between groups where appropriate. The predictive effect of low SMM on CDLT was evaluated using univariate and multivariate logistic regression analyses. Variables with a *p* value lower than 0.05 in univariate analysis were selected for inclusion in multivariate analysis. Cox proportional hazard regression analysis was used to evaluate the relationship between low SMM and overall survival (OS). Kaplan Meier curves were used to visualize overall survival.

## 3 RESULTS

Of all 279 patients predefined as having an indication for high‐dose cisplatin, 39 patients did not receive any cisplatin, and 73 patients were treated with daily cisplatin as part of a clinical study and were, thus, excluded. Six patients were treated with induction TPF (docetaxel, cisplatin, and fluorouracil), and four with weekly cisplatin, and were also excluded. In four patients, imaging quality was deemed insufficient. In total, 153 patients who were treated with three‐weekly high‐dose cisplatin were included for analysis. For the overall survival analysis, 41 patients with HPV‐positive oropharyngeal cancer were excluded, and 112 patients with HPV‐negative or unknown status were included.

### 3.1 Patient characteristics

Patient, disease, and outcome characteristics are presented in Table 1. All patients received at least 1 cycle of high‐dose cisplatin. Patients were predominantly male, current smokers, and presented with AJCC stage III or IV disease. Of note, almost 50% of all patients with oropharyngeal cancer had HPV‐related oropharyngeal cancer. Patients with HPV‐positive disease were significantly younger (57.9 vs. 60.6 years, independent sample's *t*‐test: *p* = 0.03) and had a higher BMI (25.7 vs. 23.0, independent sample's *t*‐test: *p* < 0.01) than patients with HPV‐negative disease. Approximately half of all patients completed three cycles of high‐dose cisplatin (52.9%). Two cycles of cisplatin were completed in 22.9% of patients. In 24.2% of patients, only one cycle of chemotherapy could be completed. CDLT occurred in 24.2% of patients. The most frequent reason for chemotherapy treatment termination was grade 3 toxicity, being a significant decrease in renal function in 52%, severe nausea in 9%, and infectious disease such as sepsis in 9% of patients. There were no significant differences in patients characteristics between patients with and without CDLT, apart from a mild renal function impairment prior to start of treatment with an eGFR between 60 and 70 (*p* = 0.02). Four patients had a dose reduction of cisplatin after the first cycle of 20%, resulting in a cumulative cisplatin dose of 260 mg/m^2^. There was no significant difference in the occurrence of CDLT in patients with HPV‐positive disease versus patients with HPV‐negative disease (22.0% in patients with HPV‐positive disease vs. 25.0% in patients with HPV‐negative disease, Fisher's exact test: *p* = 0.83).

**TABLE 1:** Patient, disease, and outcome characteristics

Characteristic	Total patients *n* = 153 (%)	Patients with CDLT *n* = 37 (%)	Without CDLT *n* = 116 (%)	*p* value
Gender				
Men	112 (73.2)	28 (75.7)	84 (72.4)	0.70^a^
Women	41 (26.8)	9 (24.3)	32 (27.6)	
Age at diagnosis (years)				
Mean (SD)	59.9 (6.7)	61.1 (5.9)	59.5 (7.0)	0.20^b^
Smoking				
Never	25 (16.3)	5 (13.5)	20 (17.2)	0.40^c^
Former	16 (10.5)	6 (16.2)	10 (8.6)	
Active	112 (73.2)	26 (70.3)	86 (74.1)	
Body mass index				
Mean (SD)	23.7 (4.1)	23.6 (3.8)	23.8 (4.2)	0.82^b^
ACE‐27				
0	115 (75.2)	28 (75.7)	87 (75.0)	0.20^c^
1	37 (24.2)	8 (21.6)	29 (25.0)	
2	1 (0.7)	1 (2.7)	0 (0)	
Renal function				
eGFR >70	130 (85.0)	27 (79.4)	103 (93.6)	**0.01** ^a^
eGFR 60–70	14 (9.2)	7 (20.6)	7 (6.4)	
Tumor site				
Oropharynx, HPV+	41 (26.8)	9 (24.3)	32 (27.6)	0.40^c^
Oropharynx, HPV− or unknown	51 (33.3)	12 (32.4)	39 (33.6)	
Hypopharynx	50 (32.7)	11 (29.7)	39 (33.6)	
Larynx	11 (7.2)	5 (13.5)	6 (5.2)	
T classification				
1	15 (9.8)	4 (10.8)	11 (9.5)	0.23^c^
2	46 (30.1)	10 (27.0)	36 (31.0)	
3	48 (31.4)	12 (32.4)	36 (31.0)	
4	44 (28.8)	11 (29.7)	33 (28.4)	
N classification				
0	19 (12.4)	3 (8.1)	16 (13.8)	0.53^c^
1	17 (11.1)	5 (13.5)	12 (10.3)	
2a	9 (5.9)	4 (10.8)	5 (4.3)	
2b	66 (43.1)	13 (35.1)	53 (45.7)	
2c	35 (22.9)	10 (27.0)	25 (21.6)	
3	7 (4.6)	2 (5.4)	5 (4.3)	
AJCC stage				
II	4 (2.6)	0 (0)	4 (3.4)	0.34^c^
III	66 (43.1)	14 (37.8)	52 (44.8)	
IV	83 (54.2)	23 (62.2)	60 (51.7)	
Extracapsular extension				
No	109 (71.2)	29 (78.4)	80 (69.0)	0.27^a^
Yes	44 (28.8)	8 (21.6)	36 (31.0)	
Number of cisplatin cycles				
1	37 (24.2)	37 (100)	‐	n/a
2	35 (22.9)	‐	35 (30.2)	
3	81 (52.9)	‐	81 (69.8)	
CDLT				
Absent	116 (75.8)	‐	116 (100)	n/a
Present	37 (24.2)	37 (100)		
Survival status				
Alive	99 (64.7)	21 (56.8)	78 (67.2)	0.25^a^
Deceased	54 (35.3)	16 (43.2)	38 (32.8)	

*Note*: Bold *p*‐values show significant difference.

^a^ Fisher's exact test.

^b^ Independent sample's *t*‐test.

^c^ Pearson Chi square test.

### 3.2 Low SMM as a predictor for CDLT

A sex‐specific cut‐off point for low SMM as a predictor for CDLT was formulated using an ROC curve. The AUC of the ROC curve was 0.72 for women (Mann–Whitney U test: *p* = 0.05) and 0.58 for men (Mann–Whitney U test: *p* = 0.11). The optimal cut‐off value for low SMM was 10.7 cm^2^ for women and 13.1 cm^2^ for men. Using this cut‐off, 54.9% of patients had low SMM.

### 3.3 Univariate and multivariate analyses for CDLT

Table 2 shows patient and disease characteristics of patients with low SMM and normal SMM. Patients with low SMM had a significantly lower BMI (*p* < 0.01) and a higher T stage (*p* = 0.05), and showed a trend toward a higher N stage (*p* = 0.09). There were no significant differences in terms of gender or age of patients with and without low SMM. Patients with low SMM experienced CDLT significantly more often than patients with normal SMM (35.7% vs. 10.1%; *p* < 0.01).

**TABLE 2:** Patient characteristics in patients with low and normal SMM

All patients	Patients with low SMM	Patients with normal SMM	*p* value
Characteristic	*n* = 84 (%)	*n* = 69 (%)	
Gender			
Men	64 (76.2)	48 (69.6)	0.37^a^
Women	20 (23.8)	21 (30.4)	
Age at diagnosis (years)			
Mean (SD)	59.9 (6.3)	59.8 (7.3)	0.95^b^
Smoking			
Never	14 (16.7)	11 (15.9)	0.92^c^
Former	8 (9.5)	8 (11.6)	
Active	62 (73.8)	50 (72.5)	
Body mass index			
Mean (SD)	22.1 (3.6)	25.6 (3.9)	**<0.01** ^b^
ACE‐27			
0	62 (73.8)	53 (76.8)	0.60^c^
1	21 (25.0)	16 (23.2)	
2	1 (1.2)	0 (0)	
Renal function			
eGFR >70	73 (92.4)	57 (87.7)	0.40^a^
eGFR 60–70	6 (7.6)	8 (12.3)	
Tumor site			
Oropharynx, HPV+	16 (19.0)	25 (36.2)	0.12^c^
Oropharynx, HPV− or unknown	31 (36.9)	20 (29.0)	
Hypopharynx	30 (35.7)	20 (29.0)	
Larynx	7 (8.3)	4 (5.8)	
T classification			
1	10 (11.9)	5 (7.2)	**0.05** ^c^
2	18 (21.4)	28 (40.6)	
3	27 (32.1)	21 (30.4)	
4	29 (34.5)	15 (21.7)	
N classification			
0	10 (11.9)	9 (13.0)	*0.09* ^c^
1	11 (13.1)	6 (8.7)	
2a	4 (4.8)	5 (7.2)	
2b	29 (34.5)	37 (53.6)	
2c	24 (28.6)	11 (15.9)	
3	6 (7.1)	1 (1.4)	
AJCC stage			
II	2 (2.4)	2 (2.9)	0.11^c^
III	30 (35.7)	36 (52.2)	
IV	52 (61.9)	31 (44.9)	
CDLT			
No	54 (64.3)	62 (89.9)	**<0.01** ^a^
Yes	30 (35.7)	7 (10.1)	

*Note*: Bold indicates a significant difference between groups with *p* < 0.05. Cursive indicates a *p* value <0.10.

^a^ Fisher's exact test.

^b^ Independent samples *t*‐test.

^c^ Pearson Chi square test.

In Table 3, the univariate and multivariate analyses for the occurrence of CDLT are shown. In univariate analysis, only low SMM (OR 3.75 [95% CI 1.58–8.90], *p* < 0.01) and a mild renal function impairment with an eGFR of 60–70 (OR 3.82 [95% CI 1.23–11.81], *p* = 0.02) were associated with the occurrence of CDLT. In multivariate analysis, both low SMM (OR 3.99 [95% CI 1.56–10.23], *p* = 0.01) and a mild renal function impairment (OR 5.40 [95% CI 1.57–18.65], *p* < 0.01) remained associated with the occurrence of CDLT.

**TABLE 3:** Univariate and multivariate logistic regression analyses for prediction of CDLT

	Univariate analysis		Multivariate analysis	
	Odds ratio (95% CI)	*p* value	Odds ratio (95% CI)	*p* value
Gender				
Male	Ref			
Female	0.84 (0.36–1.98)	0.70		
Age at diagnosis (years)	1.04 (0.98–1.10)	0.20		
BMI at diagnosis (kg/m^2^)	0.99 (0.90–1.08)	0.82		
Tumor site				
Oropharynx HPV+	Ref			
Oropharynx HPV− or unknown	1.09 (0.41–2.92)	0.86		
Hypopharynx	2.96 (0.72–12.00)	0.13		
Larynx	1.00 (0.37–2.72)	1.00		
AJCC stage				
II–III	Ref			
IV	1.53 (0.72–3.27)	0.27		
Renal function				
eGFR >70	Ref		Ref	
eGFR 60–70	3.82 (1.23–11.81)	**0.02**	5.40 (1.57–18.65)	**<0.01**
Low SMM				
No	Ref		Ref	
Yes	3.75 (1.58–8.90)	**<0.01**	3.99 (1.56–10.23)	**0.01**
ACE‐27				
0	Ref			
1 or 2	0.96 (0.41–2.28)	0.94		
Smoking				
No	Ref			
Former	2.40 (0.59–9.82)	0.22		
Active	1.21 (0.41–3.54)	0.73		

*Note*: Bold indicates a significant difference between groups.

### 3.4 Survival analysis

As HPV+ and HPV− HNSCC have a vastly different prognosis, we attempted to perform survival analysis separately. At the end of the follow‐up period, a total of 55 patients were deceased, of whom 53 had HPV− or unknown HPV status HNSCC (47.3% of all patients with HPV− or unknown status HNSCC), and two patients with HPV‐positive HNSCC (4.9% of patients with HPV+ HNSCC). In univariate Cox regression analysis by far, the most important prognosticator was HPV status of the tumor, with patients with HPV‐related oropharyngeal cancer having a better prognosis than other patients in this cohort (HR 0.07 [95% CI 0.02–0.31], *p* < 0.01).

There were too few events in the HPV+ patient group to conduct meaningful survival analysis. Thus, survival analysis was only performed in patients with HPV− or unknown HPV status HNSCC. Table 4 shows univariate and multivariate Cox regression analyses for OS in HPV‐negative patients or patients with unknown HPV status (*n* = 112).

**TABLE 4:** Univariate and multivariate analyses for overall survival in HPV‐negative patients

	Univariate analysis		Multivariate analysis	
	Hazard ratio (95% CI)	*p* value	Hazard ratio (95% CI)	*p* value
Gender				
Male	Ref			
Female	1.06 (0.57–1.98)	0.86		
Age at diagnosis (years)	1.02 (0.97–1.06)	0.58		
BMI at diagnosis (kg/m^2^)	0.93 (0.87–0.99)	**0.03**	0.94 (0.88–1.00)	0.07
Tumor site				
Oropharynx HPV+	Excluded^a^			
Oropharynx HPV− or unknown	Ref			
Hypopharynx	1.86 (0.74–4.69)	0.19		
Larynx	1.46 (0.81–2.61)	0.21		
AJCC stage				
II and III	Ref		Ref	
IV	3.57 (1.79–7.14)	**<0.01**	3.40 (1.69–6.81)	**<0.01**
CDLT				
No	Ref		Ref	
Yes	2.11 (1.15–3.89)	**0.02**	2.10 (1.13–3.90)	**0.02**
Low SMM				
No	Ref			
Yes	1.23 (0.71–2.16)	0.46		
ECE				
No	Ref			
Yes	1.10 (0.55–2.19)	0.80		
ACE‐27				
0	Ref			
1 or 2	0.79 (0.41–1.53)	0.48		

*Note*: Bold indicates a significant difference between groups.

^a^ HPV‐related oropharyngeal cancer: HR 0.07 (95% CI 0.02–0.31), *p* < 0.01.

**FIGURE 2:**
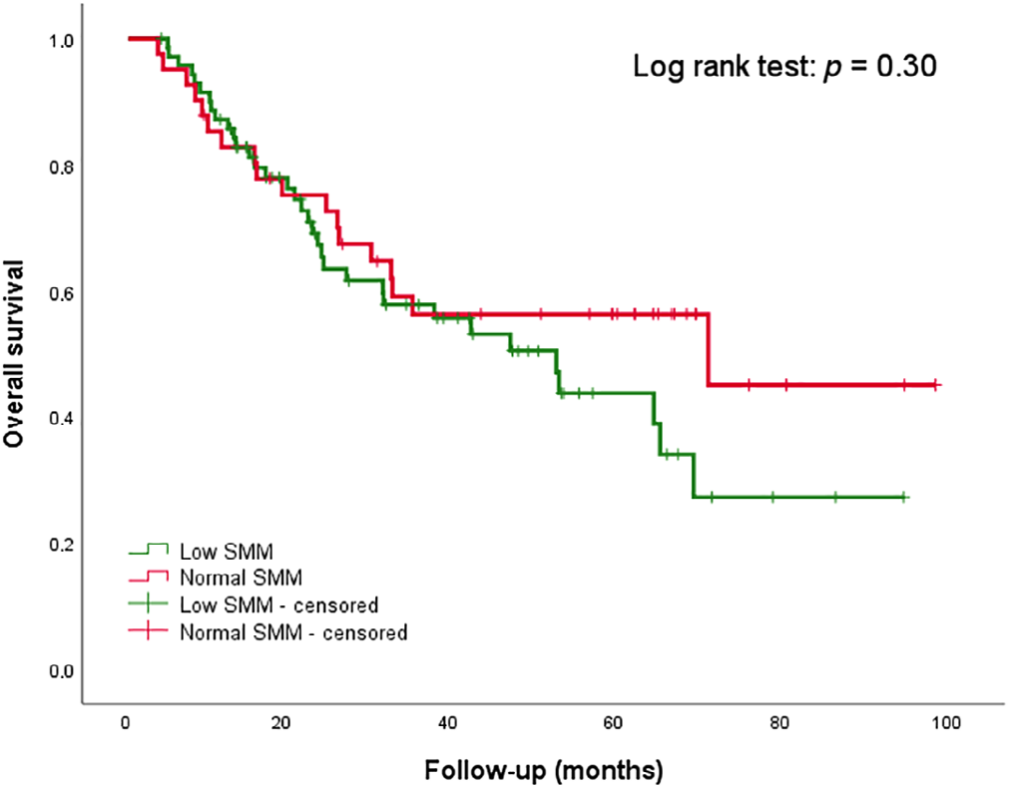
Kaplan Meier survival curve for low SMM in HPV‐negative patients

**FIGURE 3:**
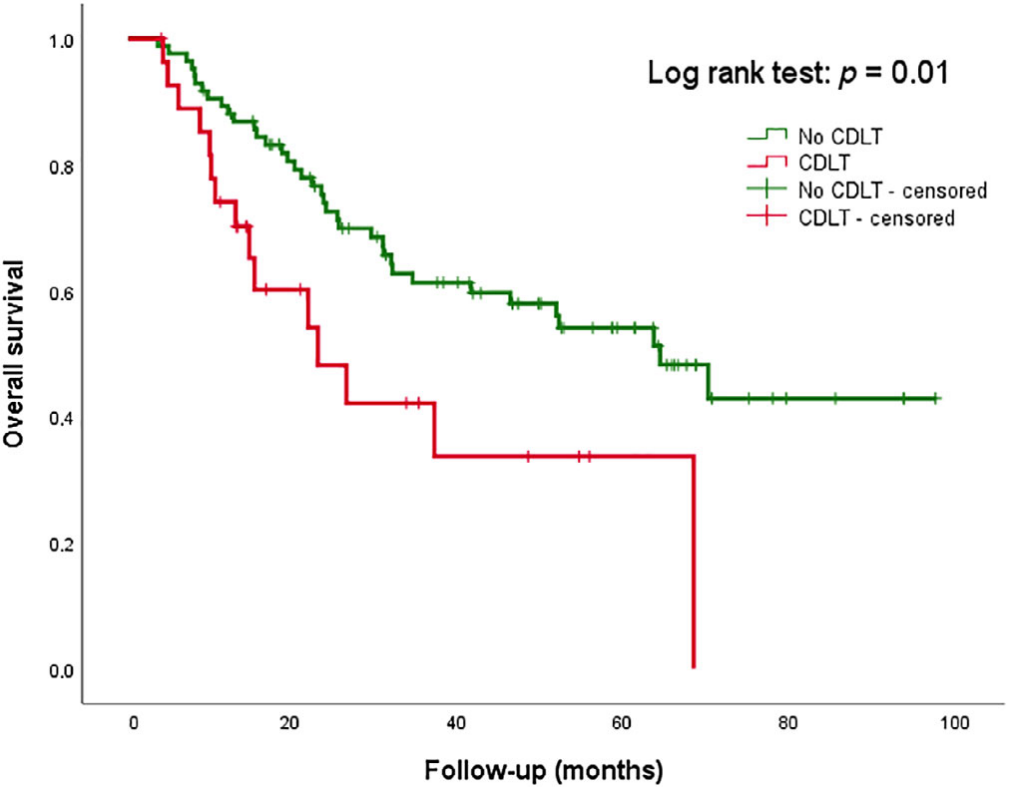
Kaplan Meier survival curve for CDLT in HPV‐negative patients

In univariate Cox regression analysis, low SMM was not a significant prognosticator (HR 1.23 [95% CI 0.71–2.16], *p* = 0.46) for OS, as visualized in Figure 2. In contrast, the occurrence of CDLT was significantly associated with a decreased OS (HR 2.11 [95% CI 1.15–3.89], *p* = 0.02), as visualized in Figure 3. Other significant prognosticators for OS were AJCC stage‐IV disease (HR 3.57 [95% CI 1.79–7.14), *p* < 0.01) and BMI (HR 0.93 [95% CI 0.87–0.99], *p* = 0.03), with a higher BMI being associated with significantly better OS. In multivariate regression analysis, only AJCC stage‐IV disease and CDLT remained significantly associated with decreased OS.

**Table:** 

	At 20 months	At 40 months	At 60 months	At 80 months
Number of events	26	44	48	52
Remaining cases	73	43	23	4

**Table:** 

	At 20 months	At 40 months	At 60 months	At 80 months
Cumulative number of events	26	44	48	52
Remaining cases	73	43	23	4

## 4 DISCUSSION

Low SMM is associated with an increase in chemotherapy‐related toxicity and CDLT in a variety of cancer types. Our study also shows this relationship in HNSCC patients treated with primary CRT with high‐dose cisplatin. Patients with low SMM had a trifold risk of experiencing CDLT compared to patients with normal SMM in this study. Although patients with low SMM did not have a decreased OS, patients who experienced CDLT did have a significantly decreased OS. This study adds to the mounting evidence that there is a clear  relationship between low SMM and the occurrence of CDLT in HNSCC patients treated with high‐dose cisplatin.^9^, ^16^, ^21^, ^22^

Platinum‐based chemotherapy is routinely used in the curative treatment of LA‐HNSCC to enhance the antitumor effect of radiation. Several treatment schemes and dosing levels are available for platinum‐based chemotherapy in HNSCC. Level 1 evidence is available for the improvement of locoregional control and overall survival with concurrent CRT with 3 three‐weekly cycles of high‐dose cisplatin at a dose level of 100 mg/m^2^ BSA.^2^ Despite irrefutable efficacy, the toxicity of treatment with high‐dose cisplatin is a well‐known problem in daily clinical practice. Early chemotherapy termination due to unacceptable toxicity occurs in approximately 30% of patients and is associated with a marked decrease in overall survival (52% vs. 72% in three‐year survival) as well as an increase in long‐term morbidity of treatment. In recent years, several large clinical trials have investigated de‐escalation strategies with weekly low‐dose cisplatin or cetuximab as radiosensitizer in HNSCC, but these trials concluded that concurrent CRT with high‐dose cisplatin remains the preferred treatment option with the highest survival benefit.^23^, ^24^, ^25^

There is an evident clinical need for improved risk assessment in patients planned for high‐dose cisplatin treatment. Several risk factors for cisplatin toxicity are already established absolute contraindications, such as a decreased renal function with an eGFR <60, severe hearing loss, or poor functional WHO status. Better knowledge on relative contraindications is needed to identify patients who may benefit from modified treatments. Low SMM is a radiological biomarker that may aid in the identification of those patients at high risk of cisplatin related toxicity that would, otherwise, not have been identified.^26^

Over the last decade, the body composition of patients with cancer has been researched extensively using diagnostic computer tomography (CT) imaging.^27^ Recent retrospective studies in a variety of cancer types have shown an association between low SMM, sometimes termed sarcopenia, and the occurrence of chemotherapeutic toxicity and CDLT.^10^ Several hypothesis have been proffered. One hypothesis behind this relationship is that most (hydrophilic) chemotherapy, including cisplatin, mainly distributes into the fat‐free body mass, of which SMM is the largest contributor.^11^, ^28^ Patients with low SMM and normal or high fat mass may receive a relatively higher dose of chemotherapy than is anticipated using a standard dosing regimen based on BSA. Previous research has shown that drug dosing based on BSA poorly predicts plasma drug concentrations of most cytotoxic drugs in individual patients, including cisplatin.^29^, ^30^ Currently, a prospective study investigating this relationship in HNSCC patients is ongoing.

It may also be that low SMM reflects an overall poorer physical functioning in patients, which is not as distinctly found as using other routinely used risk stratification methods. In recent years, there has been increased interest in the supportive care of patients with cancer undergoing chemotherapy, including increased interest in guided exercise and nutritional support during cancer treatment. A randomized controlled trial in patients with breast cancer undergoing several physical activity programs showed a positive effect on treatment tolerance and fatigue.^31^ A recently published randomized controlled trial in patients with rectal cancer undergoing neoadjuvant CRT showed a significant increase in SMM in patients who followed an exercise program during neoadjuvant chemotherapy, compared to patients who did not.^32^ A recent study in patients with breast cancer undergoing adjuvant chemotherapy did not show a difference in chemotherapy completion in patients participating in an exercise intervention, but it did show a significant decrease in hospitalization during treatment.^33^ Besides exercise and nutritional support during cancer treatment, “prehabilitation” with exercise and nutritional support prior to start of treatment is likely to increase treatment tolerance. However, limited time between diagnosis and start of treatment may decrease the ability to effectively implement a prehabilitation program in patients undergoing primary CRT.

Feasibility studies in patients with HNSCC have shown that muscle resistance training programs in patients undergoing CRT or radiotherapy are feasible and show high patient satisfaction.^34^, ^35^ Whether such interventions also provide benefit in terms of overall survival is unknown, but low SMM prior to start of treatment may be an indicator that a patient may benefit from intensified supportive care in terms of physical exercise and nutritional support. Pre‐treatment low SMM may also be used as an argument for an intended treatment de‐escalation choice, such as weekly low‐dose cisplatin, to maximize treatment adherence and cumulative cisplatin dose administered.

Several limitations to this study need to be addressed. Due to the retrospective nature of the research, not all relevant research parameters for body composition or nutritional status were measured or documented during normal clinical practice. Because of the academic nature of the tertiary referral center this study was conducted in, a relatively large percentage of patients was excluded because of a trial‐based treatment regimen (weekly or daily cisplatin).

In the present study, CDLT was defined as any toxicity resulting in a cumulative cisplatin dose of less than 200 mg/m^2^; it is generally accepted that at least a dose of 200 mg/m^2^ should be administered to be sufficiently effective.^3^, ^4^ In the previous study of Wendrich et al., CDLT was defined as any toxicity resulting in any chemotherapy dose reduction of ≥50% (e.g., due to neutropenia or nephrotoxicity), a postponement of treatment of ≥4 days (e.g., in the case of bone marrow suppression), or a definite termination of chemotherapy after the first or second cycle of therapy. Despite slightly different definitions of CDLT, the conclusions of both studies were comparable: a threefold significant higher incidence of CDLT in SMM patients (35.7% vs. 10.1% and 44.3% vs. 13.7%). In both studies, patients experiencing CDLT had a significantly lower overall survival than patients who did not.

In the current study, we decided not to use a previously published multivariate formula to calculate CSMA at the level of L3, but rather use CSMA at the level of C3 directly to assess SMM. This better allowed us to formulate a sex‐specific cut‐off point for low SMM, as is commonly done in other areas of oncological research, rather than use a single cut‐off point. It is known that women have less SMM than men.^36^ Sex is part of the previously published prediction formula for translation of CSMA at level of C3 to CSMA at level of L3, as such sex is implicitly already accounted for using this method. This choice does hinder direct comparison to our previous results. It should be noted that the incidence of low SMM as well as the trifold risk of CDLT in patients with low SMM is equal in both our previous^6^ and this current studies, and compares to results in other studies.

## 5 CONCLUSION

This study validates the previous findings that pre‐treatment low SMM is significantly associated with CDLT in LA‐HNSCC patients treated with primary CRT with high‐dose cisplatin. Pre‐treatment low SMM alone was not a prognostic factor for OS, but CDLT was. Routine SMM assessment may allow for CDLT risk assessment and identification of those patients who may benefit from treatment modifications and from interventions to increase SMM.

## Data Availability

The data that support the findings of this study are available from the corresponding author upon reasonable request.
